# Effects of acupuncture treatment on microRNAs expression in ovarian tissues from Tripterygium glycoside-induced diminished ovarian reserve rats

**DOI:** 10.3389/fgene.2022.968711

**Published:** 2022-09-21

**Authors:** Ge Lu, Yao-yao Zhu, Hong-xiao Li, Yao-li Yin, Jie Shen, Mei-hong Shen

**Affiliations:** ^1^ College of Acupuncture Moxibustion and Tuina, Nanjing University of Chinese Medicine, Nanjing, China; ^2^ Key Laboratory of Acupuncture and Medicine Research of Ministry of Education, Nanjing University of Chinese Medicine, Nanjing, China

**Keywords:** acupuncture, diminished ovarian reserve, microRNA, oxidative stress, sequence

## Abstract

Acupuncture is widely used to improve ovarian function. Previously, we demonstrated that acupuncture can improve oxidative stress in rats with tripterygium glycoside tablet suspension (TG)-induced diminished ovarian reserve (DOR). Herein, we aimed to explore the antioxidation mechanism of acupuncture for ameliorating the ovarian reserve in DOR rats. We performed microRNA sequencing and bioinformatics analysis to screen differentially expressed miRNAs (DE miRNAs) in ovarian tissues. In total, 1,172 miRNAs were identified by miRNA sequencing, of which 28 DE miRNAs were detected (including 14 upregulated and 14 downregulated) in ovarian tissues from the acupuncture group when compared with the DOR model rats. Based on functional enrichment analysis, the target genes of DE miRNAs were significantly enriched in GO-biological process (BP) terms associated with biological processes, positive regulation of transcription by RNA polymerase II, signal transduction, regulation of transcription, DNA-templated processes, and oxidation–reduction processes. In the Kyoto Encyclopedia of Genes and Genomes analysis, the main pathways were the MAPK signaling pathway, hepatitis B, proteoglycans in cancer, human cytomegalovirus infection, and the Ras signaling pathway. Finally, reverse transcription-quantitative PCR results confirmed that rno-miR-92b-3p, mdo-miR-26b-5p_R+1_1ss10TC, and bta-miR-7857-3p_R-1 were downregulated in the acupuncture group. The results revealed the impact of acupuncture on miRNA profiling of ovarian tissues from DOR rats, suggesting that rno-miR-92b-3p, mdo-miR-26b-5p_R+1_1ss10TC, and bta-miR-7857-3p_R-1 might provide relevant cues to relieve DOR-mediated oxidative stress.

## 1 Introduction

Diminished ovarian reserve (DOR) often occurs before the age of 40 years in women and is characterized by a decline in reproductive capacity, especially a decrease in the number and quality of oocytes, accompanied by menstrual disorders, hot flashes, and emotional abnormalities, which seriously impacts the physical and mental health of patients and reduces their quality of life (Quinn and Cedars, 2018). DOR is considered the early stage of premature ovarian insufficiency or premature ovarian failure (POF). Studies have shown that DOR can progress to POF in approximately 1–6 years (Greene et al., 2014). In addition to genetic abnormalities, autoimmune disorders, and medical factors (Devine et al., 2015), chemotherapy is a common cause of DOR (Hopeman et al., 2021). Female patients of reproductive age who undergo chemotherapy are at high risk for ovarian reserve damage (Bedoschi et al., 2016). Therefore, protecting the ovarian reserve function during the early stage of chemotherapeutic intervention is necessary in order to delay the decline in fertility and prevent POF.

Acupuncture, a form of traditional Chinese medicine, is considered a beneficial treatment for combating DOR. Prospective cohort studies have reported that acupuncture could regulate menstrual cycles and promote pregnancy without infection and organ injury in patients with DOR (Li et al., 2017; Yang and Yang, 2020). In addition, animal studies have confirmed that acupuncture and moxibustion can improve ovarian function in DOR rats (Zhang et al., 2019; Lu et al., 2021). Compared with hormone replacement therapy (Liu et al., 2019), assisted reproductive technology (Vassard et al., 2021; Stern et al., 2022), and stem cell transplantation (Tauchmanovà et al., 2007), acupuncture shows fewer side effects (Li et al., 2017). Several mechanisms are known to account for the pathogenesis of DOR. Oxidative stress plays a key role in granulosa cell apoptosis and follicular atresia (Zou et al., 2020). Hence, several studies have focused on the balance between DOR and oxidative stress (Soylu Karapinar et al., 2017; Wang et al., 2018; Lim et al., 2020). Consistent with these results, we previously reported that DOR rats treated with tripterygium glycoside tablet suspension (TG) exhibit diminished anti-oxidative ability; this effect was reversed by acupuncture or moxibustion, as evidenced by increased superoxide dismutase (SOD) activity and decreased malondialdehyde (MDA) content in the serum post-intervention (Fang et al., 2019; Wang et al., 2021).

MicroRNAs (miRNAs) are small non-coding RNAs, 20–25 nucleotides in length. Considering the critical role of miRNAs in regulating gene expression and interactive signaling events (Flynt and Lai, 2008), their involvement in cumulus and granulosa cells has been demonstrated (Chen et al., 2017; Hong et al., 2018; Kim et al., 2021a; Wei et al., 2021). Despite the widespread application of miRNA sequencing (Raza et al., 2022), the effect of acupuncture on miRNA profiling in DOR rats has not been fully elucidated.

To increase our understanding of DOR, we analyzed ovarian miRNA profiles of DOR rats. Herein, we reveal that acupuncture could improve the ovarian reserve in TG-induced DOR rats by regulating miRNA expression. In addition, from our results, we identified 14 upregulated and 14 downregulated miRNAs in the acupuncture-treated group. We confirmed rno-miR-92b-3p, mdo-miR-26b-5p_R+1_1ss10TC, and bta-miR-7857-3p_R-1 as key regulators following acupuncture intervention. Our findings can establish an important theoretical basis for diagnosing and treating DOR.

## 2 Materials and methods

### 2.1 Animals

Eight-week-old female Sprague–Dawley rats (190 ± 10 g) were purchased from Shanghai Xipuer-Bikai Lab Animal Co. Ltd. (license number: SCXK (Hu) 2018-0006). Five rats were housed in standard polypropylene cages and maintained under the appropriate temperature (24–26°C) and humidity (50–60%) with 12:12 h light–dark cycles. Water and food were available ad libitum.

### 2.2 Ethical approvals

The experimental protocol was approved by the Animal Ethics Committee of Nanjing University of Chinese Medicine Laboratory Animal Center (No. 201804A014).

### 2.3 Diminished ovarian reserve model establishment and acupuncture protocol

After a 10-day screening period, we selected only 18 rats with normal estrous cycles, which were divided into 3 groups using a randomized digital table (Supplementary Table S1): the control group (CON; *n* = 6), the DOR model group (*n* = 6), and the acupuncture group (ACU; *n* = 6). Based on a previous study (Fang et al., 2019), TGs (Hunan Qianjin Co., Ltd., Hunan, China) were used to establish the rat model of DOR. TG (50 mg/kg/day) was administered by intragastric administration for 14 consecutive days to establish DOR rats. Rats in the CON group were administered physiological saline (10 ml/kg/day) via gavage. The ACU group rats received acupuncture therapy with needles (0.20 mm × 13 mm; Suzhou Medical Supplies Co., Ltd., Suzhou, China) after intragastric administration of TGs. Acupuncture was applied to two sets of acupoints on alternate days, “Guan Yuan” (CV4) and “Zhong Wan (CV12),” and the other was bilateral “Shen Shu (BL23).” The needles were retained for 10 min at each acupoint. All interventions lasted for 14 days.

On day 15, the bilateral ovaries of each rat were excised. The left ovaries of each group were fixed in 4% neutral buffered paraformaldehyde for subsequent histopathological studies. Three right ovaries were used for miRNA sequencing, and the other three right ovaries were used for miRNA quantification.

### 2.4 Estrous cycle examination

To examine the estrous cycle of each rat, vaginal swabs were collected at 8:30 a.m. daily. The estrous cycles in normal adult rats averaged 4–5 days, which consisted of four phases. During diestrus, a large number of leukocytes could be observed under a light microscope (Olympus CX21, Japan); after diestrus, proestrus, characterized by a few leukocytes and a large number of nucleated epithelial cells, was observed; subsequently, the estrus phase exhibited a large number of squamous enucleated epithelial cells; finally, three types of cells were present during metestrus (Westwood, 2008).

### 2.5 Hematoxylin and eosin staining

Ovaries were fixed in 4% neutral buffered paraformaldehyde, dehydrated in ethanol, and embedded in paraffin. The paraffin blocks were sliced into 6-μm sections and then stained with HE. Every 10th section of each ovary was used to estimate the number of follicles. Only follicles with nuclei in the oocytes were counted to avoid repeated counting. Follicles were classified as primordial, primary, secondary, antral, and atresia, as previously described. Raw counts from each section of one ovary were converted to the total number of follicle stages: raw count × 10. Histopathological images were obtained using a U-ND6-2 fluorescence multifunctional microscope (Olympus, Japan).

### 2.6 RNA extraction, cDNA library construction, and miRNA expression profiling analysis

Three right ovaries from each group were used for miRNA sequencing. Based on the protocol, the total RNA of ovarian tissue from the CON, DOR, and ACU groups was extracted using TRIzol. Sequencing was performed strictly based on standard steps provided by Illumina Inc., including library construction and sequencing tests. TruSeq Small RNA Sample Prep Kit (Illumina, San Diego, CA, United States) was used for miRNA library construction. Subsequently, the constructed miRNA library was sequenced on an Illumina HiSeq 2000/2500 (Illumina, San Diego, CA, United States) with a single/paired-end 1- to 150-bp read run. ACGT101-miR (developed independently by LC Sciences, Houston, Texas, United States) was used for the data analysis. First, the 3′-adaptor and garbage sequences were removed to obtain clean data. Subsequently, sequences ≤18 nt in length were cleaned. Finally, the measured sequences were filtered using mRNA, RFam, and Repbase databases, and miRNAs were identified by comparing the precursor and genome. Differentially expressed miRNAs (DE miRNAs) were compared between the CON, DOR, and ACU groups based on the criteria of |log2 fold change (FC)| >1 and *p*-value < 0.05. The prefixes of miRNAs such as rno, mdo, and bta only indicate that the miRNAs were first discovered in the corresponding species. The tail suffixes after -3p and -5p are based on the miRBase database specification to clarify the position of the miRNA at the end of the precursor’s arm and the form of the heterodimer; for example, R+1 in mdo-miR-26b-5p_R+1_1ss10 TC indicates an extra base at the right end of the miRNA included in miRBase, and 1ss10 TC indicates a C substitution at the 10th base T (ss, substitution).

### 2.7 miRNA target gene prediction and enriched analysis

The target genes for significant miRNAs in the ACU and DOR groups were predicted using TargetScan (Whitehead Institute for Biomedical Research, Cambridge, United States) (Kim et al., 2021b) and miRanda (Memorial Sloan-Kettering Cancer Center, New York, United States) (Li et al., 2022). Target genes with context score percentiles ˂ 50 (by TargetScan) and maximum energy ˃ −10 (by miRanda) were cleaned up. Only overlapping miRNAs filtered by these two databases were used as final target genes for further analysis.

Gene Ontology (GO) function annotations and the Kyoto Encyclopedia of Genes and Genomes (KEGG) were used to determine the biological implication of target genes. GO consisted of three categories: characterizing the molecular function (MF) of genes, cellular component (CC), and biological process (BP). KEGG was used to obtain pathways for each target gene, and the compiled pathways were mapped to differentially expressed genes. A *p*-value of <0.05 was deemed statistically significant.

### 2.8 miRNA-Gene Ontology and miRNA-Kyoto Encyclopedia of Genes and Genomes interaction network analysis

To further understand the correlations between target genes of miRNAs and functional annotations, the miRNA-GO and miRNA-KEGG networks were constructed using Cytoscape software (version 3.6.0). We evaluated the top 10 biological processes in the GO analysis and the KEGG ranked by the degree method in the network. The higher the degree of GO and KEGG, the more important the role they played. Given the role of oxidative stress in DOR and our previous study, terms related to redox were extracted to evaluate the target genes.

### 2.9 Reverse transcription-quantitative PCR (RT-qPCR)

qRT-PCR analysis was performed to validate miRNA sequencing data. Total RNA was extracted from the ovaries of the CON, DOR, and ACU groups using TRIzol (Invitrogen, Carlsbad, CA, United States) to synthesize template DNA using an miRNA First-Strand cDNA Synthesis Kit (tailing reaction) (Shanghai Bioengineering Technology Service Co., Ltd., Shanghai, China) or Hifair^®^Ⅱ First-Strand cDNA Synthesis Kit (gDNA digester plus) (Yeasen Biotechnology Co., Ltd., Shanghai, China). The 3-step program was performed with the Stratagene Mx3000P real-time PCR system (Agilent Technologies Co. Ltd., California, United States) using relative quantification in a 20-μl setup, using 2 μg total RNA. The results were analyzed using a comparative cycle threshold (CT) method and calculated from the △△CT values using the formula: 2^−△△CT^. The relative quantitation values of rno-miR-92b-3p, rno-miR-206-3p, mdo-miR-26-5p_R+1_1ss10TC, bta-miR-7857-3p_R-1, rno-miR-219a-2-3p_1ss10GC, and PC-3p-66859_94 were normalized to the level of the U6 reference gene **(** Supplementary Table S2 **)**.

### 2.10 Statistical analysis

The miRNA sequencing data were analyzed using bioinformatics methods as described previously. Quantitative data were analyzed by one-way analysis of variance (ANOVA) among multiple groups, followed by the least significant difference test for comparing every two groups. The Kruskal–Wallis test was used for non-normally distributed data, and Tamhane’s T2 test was used for nonhomogeneity of variance. The results of the quantitative data are expressed as mean ± standard deviation (SD). A *p*-value of <0.05 was deemed statistically significant. All statistical analyses were performed using Statistical Package for the SPSS (version 26.0; IBM Corp., Armonk, NY, United States). Bioinformatics analysis was performed using the OmicStudio tools at https://www.omicstudio.cn/tool.

## 3 Results

### 3.1 TG-induced diminished ovarian reserve rats showed distinct phenotypes and miRNA expression

#### 3.1.1 TG-induced irregular estrous cycles and decreased ovarian reserve in rats

**FIGURE 1:**
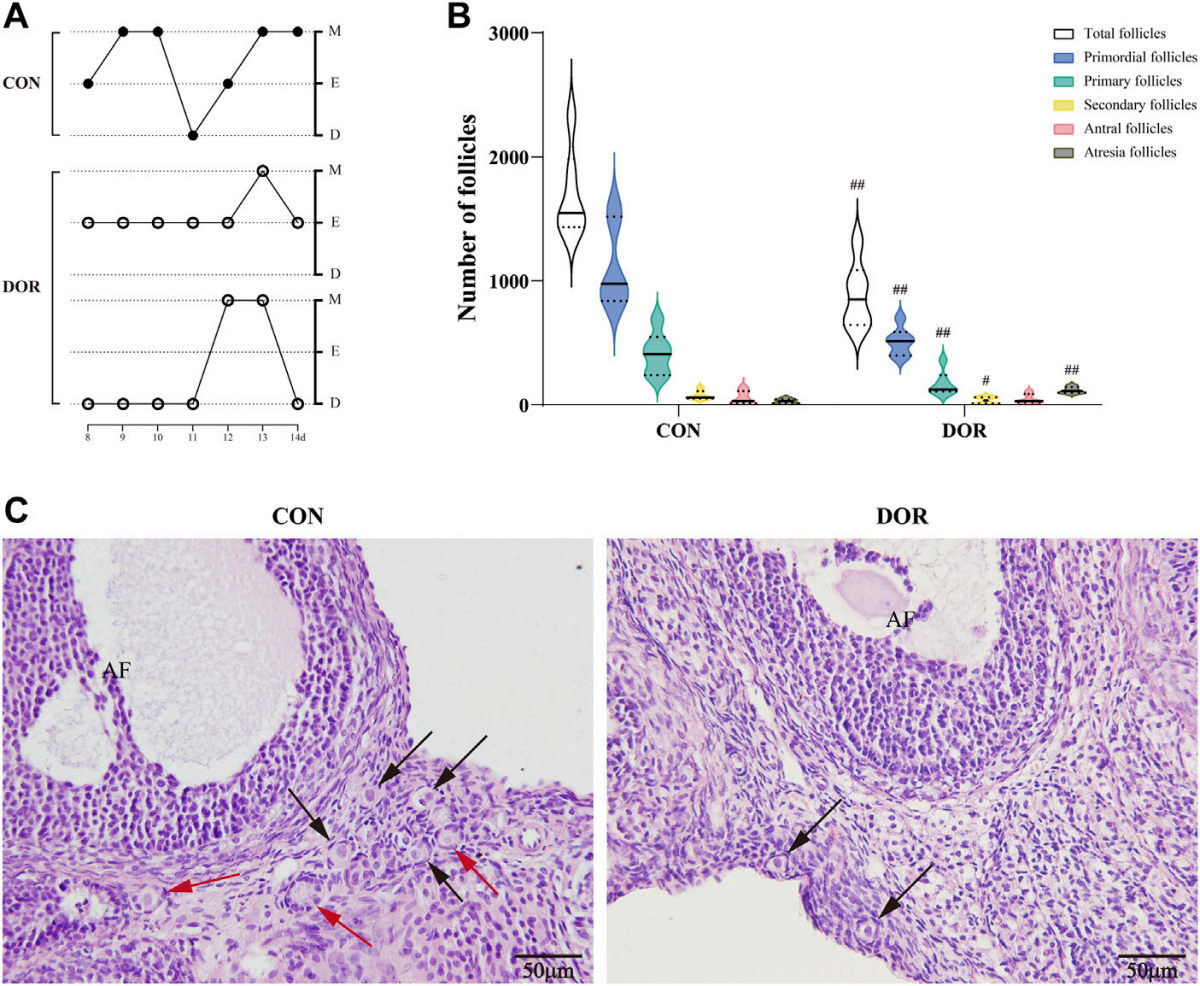
DOR rats exhibit disordered estrous cycles and a decline in ovarian reserve. **(A)** Estrous cycles from day 8 to 14. **(B)** Number of follicles in the CON and DOR groups; compared with CON, ^##^ *p* < 0.01, #*p* < 0.05. **(C)** Representative photographs of HE staining of ovarian slices. Black arrows indicate primordial follicles, and red arrows indicate primary follicles; AF, antral follicles; scale bar = 50 µm. *n* = 6. CON, control group; DOR, diminished ovarian reserve; HE, hematoxylin and eosin.

During the 14-day experimental period, the rats’ estrous cycles were examined from days 8 to 14. We detected the presence of two different types of disordered estrous cycles in the DOR group: prolonged estrous stage (≥3 days) and successive diestrus without the estrous phase (Figure 1A). Next, we examined the ovarian reserve in the DOR group. Compared with the CON group, the total number of follicles, primordial follicles, primary follicles, and secondary follicles were significantly decreased (*p* < 0.01, *p* < 0.01, *p* < 0.01, and *p* < 0.05) in rats in the DOR group, accompanied by a dramatic increase in the number of atretic follicles (*p* < 0.01) **(** Figure 1B). The ovarian sections exhibited few primordial follicles around the developed follicles in the corpus luteum (Figure 1C).

#### 3.1.2 Diminished ovarian reserve rats expressed various miRNA profiles

**FIGURE 2:**
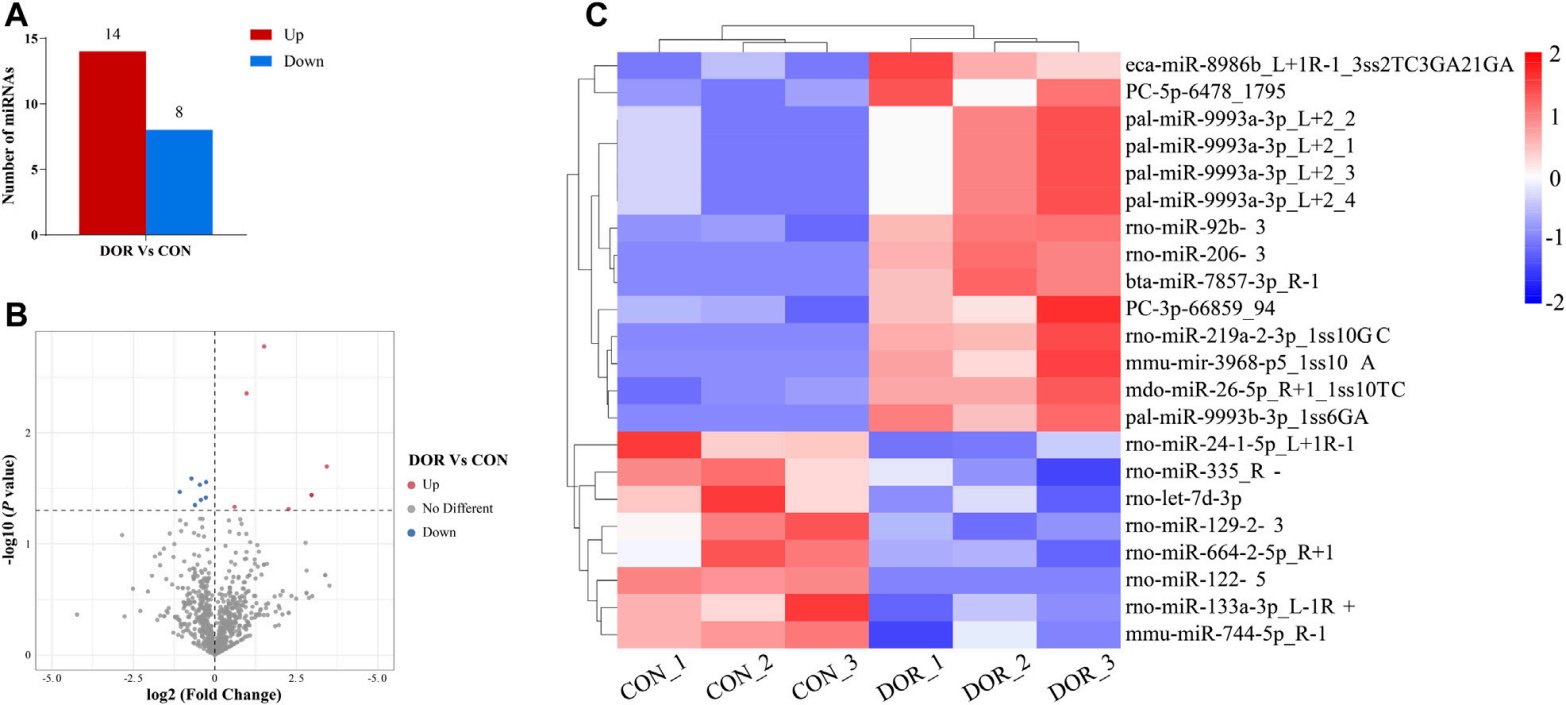
Analysis of DE miRNAs in the CON and DOR groups. **(A)** The number of upregulated and downregulated DE miRNAs was identified (*p* < 0.01). **(B)** Volcano plot showing DE miRNAs (*p* < 0.05). **(C)** Heatmap showing the relative expression of DE miRNAs (*p* < 0.05, log2 (FC) ≥ 1 or <=−1; red: high expression; blue: low expression). CON, control group; DOR, diminished ovarian reserve; DE miRNAs, differentially expressed miRNAs.

Overall, 1,188 and 1,281 miRNAs were detected in the ovarian tissues of the CON and DOR rats, respectively. Compared with the CON group, a total of 1,162 miRNAs were identified in the DOR rats using high-throughput miRNA sequencing, of which 22 were DE miRNAs, including 14 upregulated and 8 downregulated (Supplementary Table S3, Figures 2A–C).

### 3.2 Acupuncture plays a protective role against diminished ovarian reserve by regulating miRNA

#### 3.2.1 Acupuncture ameliorated ovarian function in diminished ovarian reserve rats

**FIGURE 3:**
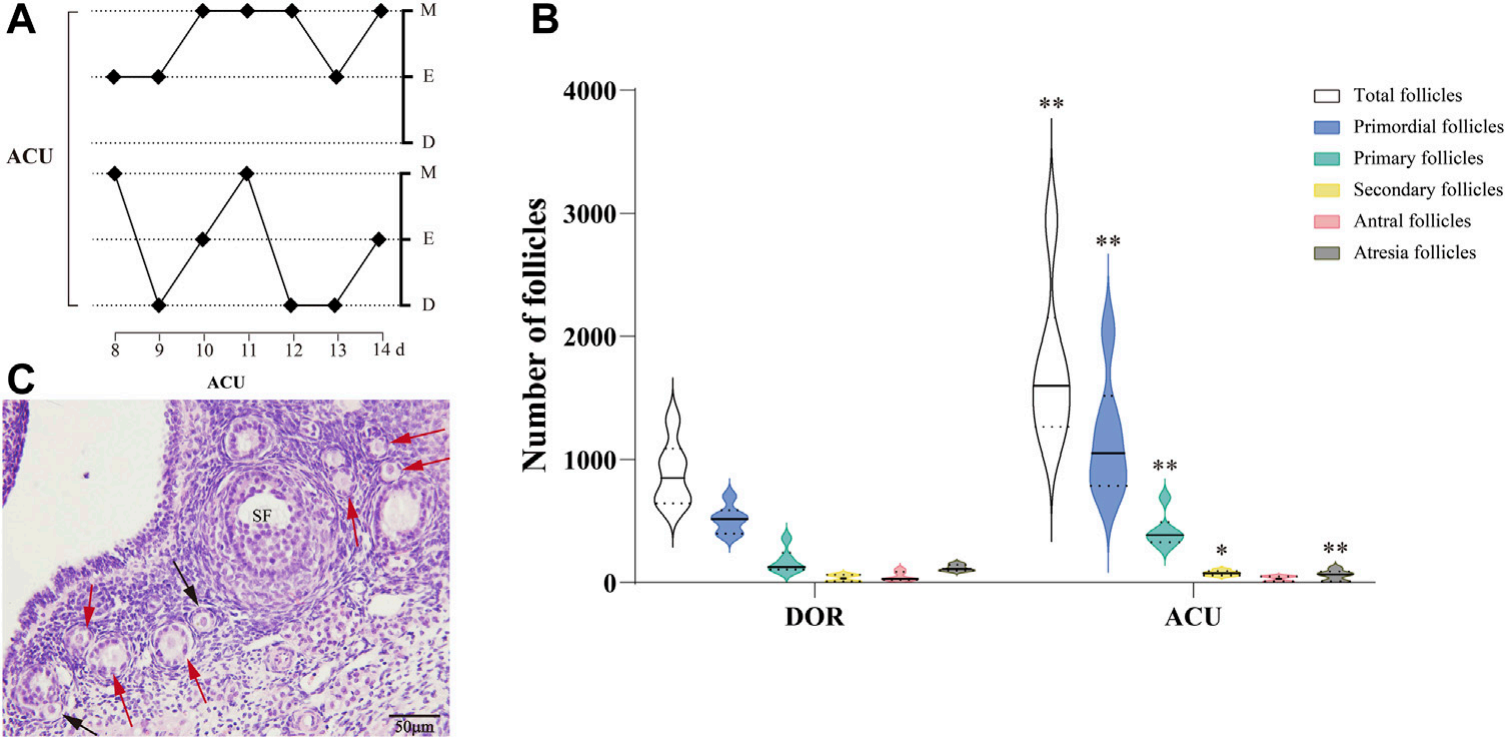
Acupuncture improves the ovarian reserve in DOR rats. **(A)** Estrous cycles in the ACU group. **(B)** Follicle counts in the DOR and ACU groups. **(C)** Representative images of HE-stained ovarian sections. Black arrows indicate primordial follicles, and red arrows indicate primary follicles. Scale bar = 50 μm. Compared with DOR, ^**^ *p* < 0.01, ^*^ *p* < 0.05; *n* = 6. SF, secondary follicles; CON, control group; DOR, diminished ovarian reserve.

Vaginal cytology revealed that acupuncture restored the estrous phases and regulated the estrous cycles in the ACU group **(** Figure 3A **)**. In addition, the follicle counts showed that acupuncture could improve the ovarian reserve in DOR rats **(** Figure 3C). Specifically, the total number of follicles and the number of primordial, primary, and secondary follicles were significantly increased in the ACU group (*p* < 0.01). Furthermore, the ACU group exhibited a significant increase in the number of atretic follicles (*p* < 0.05) **(** Figure 3B).

#### 3.2.2 Acupuncture altered ovarian miRNA expression in diminished ovarian reserve rats

**FIGURE 4:**
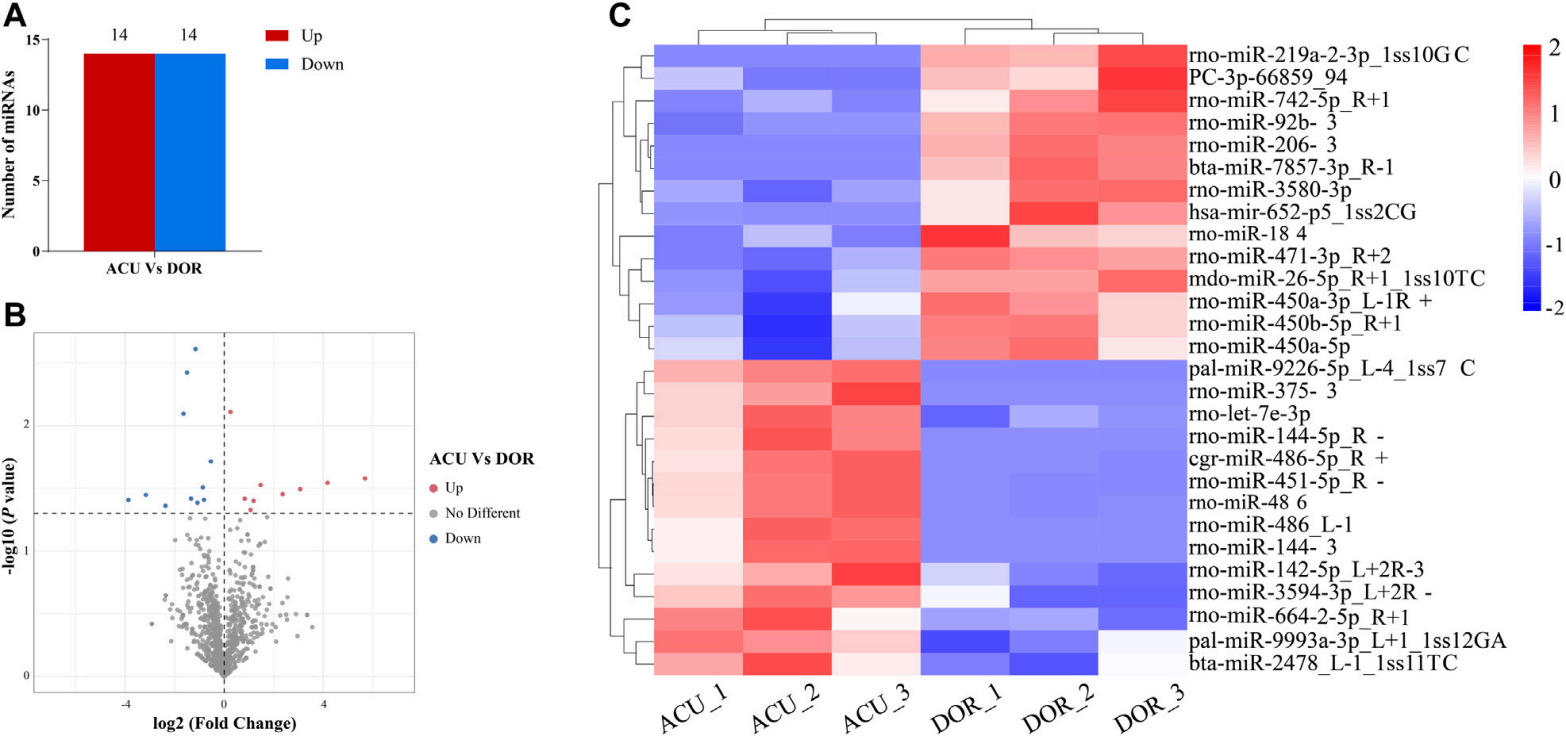
Acupuncture alters miRNA expression in DOR rats. **(A)** The number of upregulated and downregulated DE miRNAs was identified (*p* < 0.01). **(B)** Volcano plot showing DE miRNAs (*p* < 0.01). **(C)** Heatmap showing the relative expression of DE miRNAs (*p* < 0.05, log2 (FC) ≥ 1 or <=−1; red: high expression; blue: low expression). ACU, acupuncture group; CON, control group; DOR, diminished ovarian reserve; DE miRNAs, differentially expressed miRNAs.

In total, 1,214 miRNAs were identified in the ACU group. Of these, 1,172 miRNAs differed significantly between the ACU and DOR groups, and 28 were DE miRNAs, including 14 upregulated and 14 downregulated (Supplementary Table S4; Figures 4A–C).

#### 3.2.3 Functional enrichment analysis of target genes for differentially expressed miRNAs between ACU and diminished ovarian reserve rats

**FIGURE 5:**
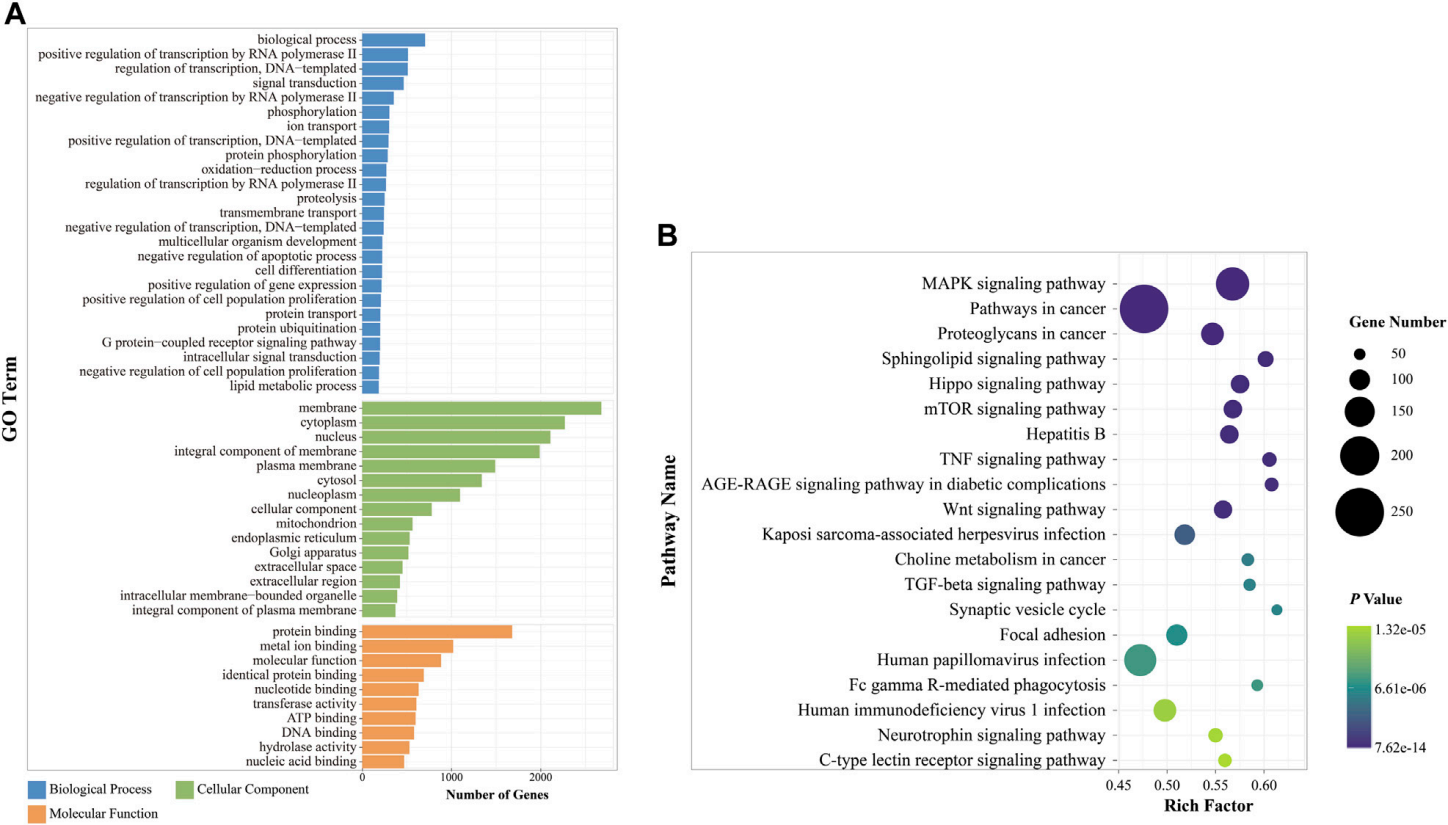
Functional enrichment analysis of DE miRNAs between the ACU and DOR groups. **(A)** GO annotation of DE miRNAs after acupuncture (*p* < 0.01). The *x*-axis indicated the number of miRNAs in each term, and the *y*-axis on the left indicated the enriched GO term. Different colors represented different GO terms. **(B)** KEGG enrichment of DE miRNAs after acupuncture. The top 20 terms are displayed (*p* < 0.01). The size of each dot is based on the enrichment of DE miRNAs. GO, Gene Ontology; KEGG, Kyoto Encyclopedia of Genes and Genomes. ACU, acupuncture group; CON, control group; DOR, diminished ovarian reserve; DE miRNAs, differentially expressed miRNAs.

Overall, 7,376 genes were identified as target genes, with 14 upregulated and 14 downregulated DE miRNAs **(** Supplementary Table S4 **)**. To evaluate potential functions, GO analysis of target genes was performed. The target genes were classified into three ontologies: 9,602 biological processes, 1,405 cellular components, and 2947 MFs (Supplementary Material). In total, 50 significantly enriched GO terms were found in the histogram (Figure 5A). In the case of biological processes, target genes were found to be involved in DNA and RNA transcription, signal transduction, redox, histone modifications, cell development, and cell metabolism. Of the target genes, 286 were clustered into the “oxidation–reduction process.” Most cellular components were focused on the membrane, cytoplasm, and nucleus, and a few genes were related to the organelle. Based on molecular function, target genes converged on enzyme activity, protein, and molecule binding.

According to the KEGG pathway analysis, 320 pathways (Supplementary Material) were annotated (Figure 5B). Among the top 20 enriched pathways, one-quarter were related to cellular pathways, including the sphingolipid signaling pathway, TGF-beta signaling pathway, Hippo signaling pathway, mTOR signaling pathway, MAPK signaling pathway, and Wnt signaling pathway. Three of these were associated with immune system regulation: the TNF signaling pathway, Fc gamma R-mediated phagocytosis, and C-type lectin receptor signaling pathway; others were clustered into the nervous system, viral infection, and cancer development.

**FIGURE 6:**
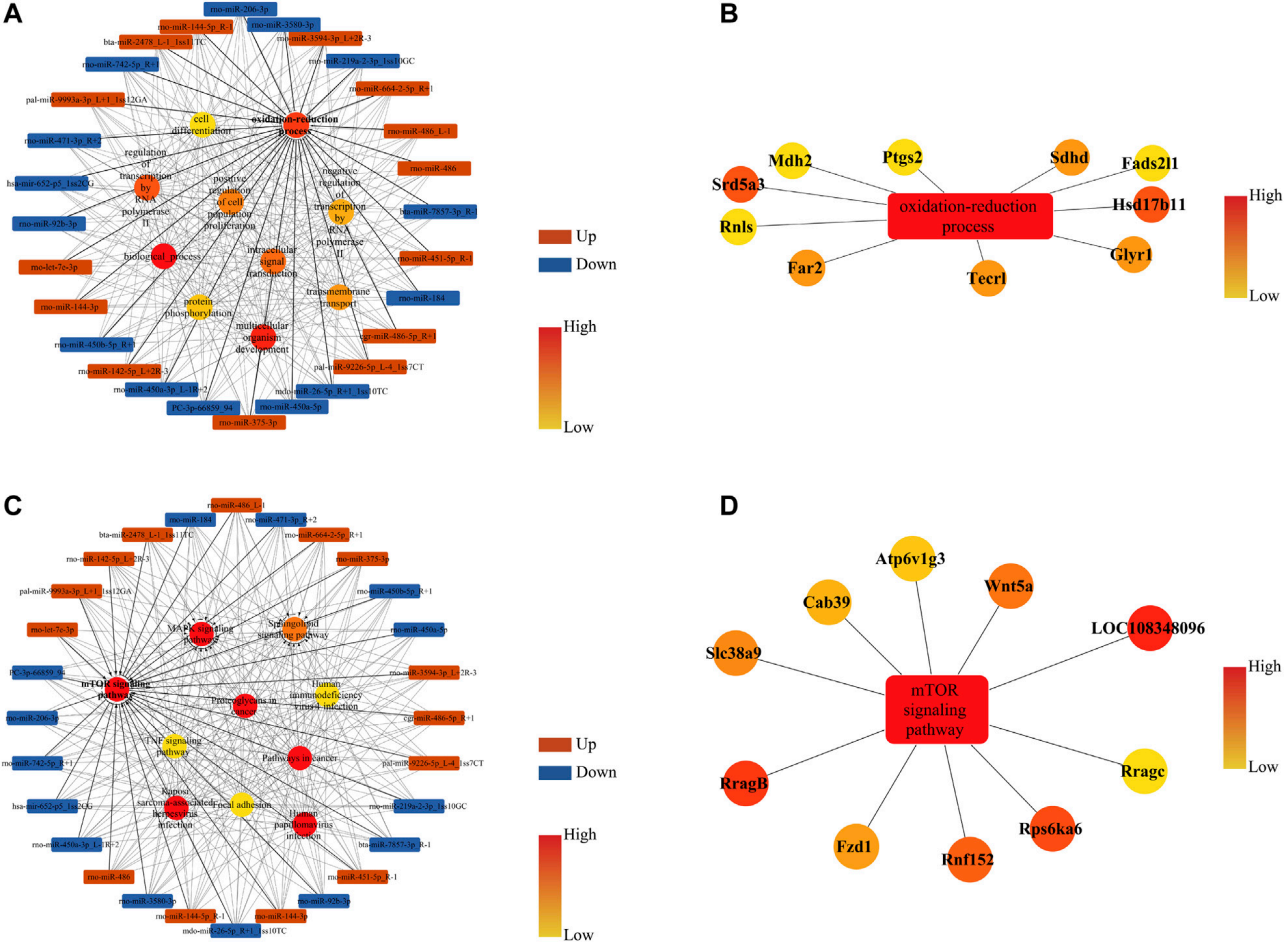
miRNA-GO and miRNA-KEGG networks of DE miRNAs and their corresponding target genes (mRNAs) in rat ovaries after acupuncture intervention compared with DOR rats. **(A)** Box nodes denote the DE miRNAs, circle nodes denote the first 10 degree BPs, and edges show the interactions between miRNAs and GOs. **(B)** Extracted from **A**, box nodes indicate BP, circle nodes indicate the first 10 target genes, and edges indicate the interactions between genes and GO terms. **(C)** Box nodes denote the DE miRNAs, circle nodes denote the first 10 degree KEGG, and edges show the interactions between miRNAs and pathways. **(D)** Extracted from **C**, box nodes represent pathways, circle nodes represent the first 10 target genes, and edges show the interactions between genes and pathways. ACU, acupuncture group; BP, biological process; CON, control group; DOR, diminished ovarian reserve; DE miRNAs, differentially expressed miRNAs; GO, Gene Ontology; KEGG, Kyoto Encyclopedia of Genes and Genomes.

The miRNA-GO network interconnected 28 DE miRNAs, and a significant BP was established in the GO analysis. The top 10 BP terms regulated by DE miRNAs were biological process, oxidation–reduction process, multicellular organism development, regulation of transcription by RNA polymerase Ⅱ, intracellular signal transduction, positive regulation of cell population proliferation, transmembrane transport, negative regulation of transcription by RNA polymerase Ⅱ, protein phosphorylation, and cell differentiation (Figure 6A). Considering the regulatory effect of acupuncture on oxidative stress in TG-induced DOR rats (Fang et al., 2019), the oxidation–reduction process was extracted to analyze the target genes. Our results revealed that Hsd17b11, Srd5a3, Sdhd, Far2, Tecrl, Glyr1, Fads211, Ptgs2, Mdh2, and Rnls were the key genes in the oxidation–reduction process (Figure 6B).

The miRNA-KEGG network interconnected 28 DE miRNAs, and significant pathways were identified. The top 10 pathways regulated by DE miRNAs were the mTOR signaling pathway, MAPK signaling pathway, proteoglycans in cancer, pathways in cancer, human papillomavirus infection, Kaposi sarcoma-associated herpesvirus infection, sphingolipid signaling pathway, TNF signaling pathway, human immunodeficiency virus infection, and focal adhesion (Figure 6C). Our study demonstrated that restoring the PI3K/AKT signaling pathway could improve the ovarian reserve in DOR rats (Li et al., 2022), and mTOR is a key downstream target of the PI3K/AKT pathway. Therefore, we further analyzed the mTOR signaling pathway to identify target genes. The results suggested that RragB, LOC108348096, Rps6ka6, Wnt5a, Rnfl52, Slc38a9, Fzd6, Cab39, Atp6v1g3, and Rragc play pivotal roles in regulating the mTOR signaling pathway (Figure 6D).

#### 3.2.4 qRT-PCR validation confirmed differentially expressed miRNAs in the ACU group

**FIGURE 7:**
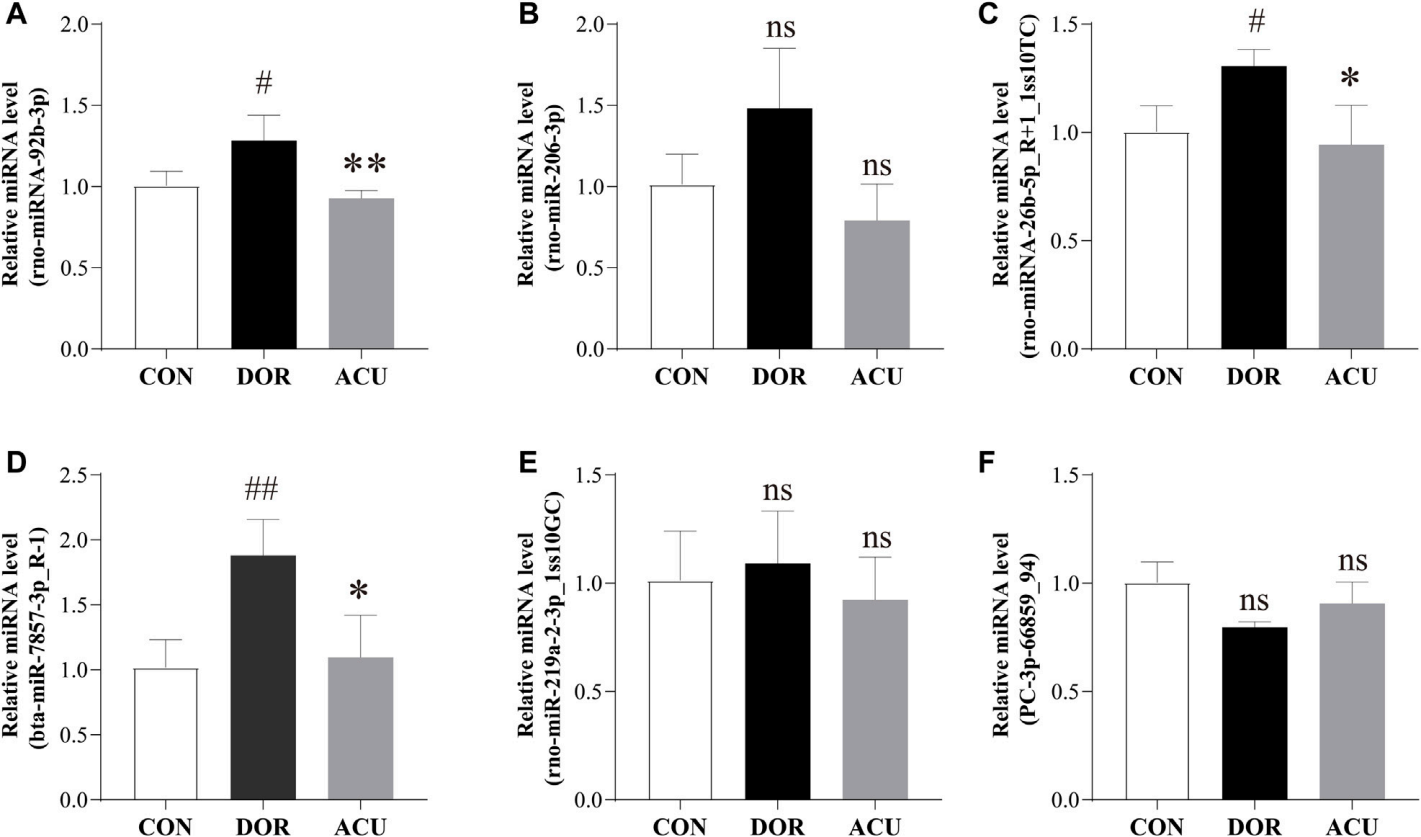
Detection of selected differentially expressed miRNAs. Expressions of **(A)** rno-miR-92b-3p, **(B)** rno-miR-206-3p, **(C)** mdo-miR-26b-5p_R+1_1ss10TC, **(D)** bta-miR-7857-3p_R-1, **(E)** rno-miR-219a-2-3p_1ss10GC, and **(F)** PC-3p-66859_94 were determined in the ovaries of CON, DOR, and ACU groups by RT-qPCR. Data are presented as mean ± standard deviation (SD). Compared with CON, ^##^ *p* < 0.01, ^#^ *p* < 0.05; compared with DOR, ^**^ *p* < 0.01, ^*^ *p* < 0.05; not significant, ns; *n* = 3. CON, control group; DOR, diminished ovarian reserve; ACU, acupuncture group.

Overall, 21 DE miRNAs overlapped between “ACU vs. DOR” and “DOR vs. CON.” Subsequently, six DE miRNAs, namely, rno-miR-92b-3p, rno-miR-206-3p, mdo-miR-26b-5p_R+1_1ss10TC, bta-miR-7857-3p_R-1, rno-miR-219a-2-3p_1ss10GC, and PC-3p-66859_94, in overlapping areas with | log2 (fold change) | ≥1, were selected for further analysis. Compared with the CON group, rno-miR-92b-3p, mdo-miR-26b-5p_R+1_1ss10TC, and bta-miR-7857-3p_R-1 levels were significantly increased in the DOR group (*p* < 0.05, *p* < 0.05, and *p* < 0.01, respectively). Compared with the DOR group, rno-miR-92b-3p, mdo-miR-26b-5p_R+1_1ss10TC, and bta-miR-7857-3p_R-1 were decreased in the ACU group (*p* < 0.01, *p* < 0.05, and *p* < 0.05, respectively) (Figure 7).

## 4 Discussion

In the present study, we established a DOR rat model via intragastric administration of TGs. Characterization of sex hormones in the serum, including estradiol (E2), follicle-stimulating hormone, and anti-Müllerian hormone (AMH), was confirmed in our previous studies (Fang et al., 2019; Lu et al., 2021; Wang et al., 2021; Li et al., 2022). In addition, TG-induced DOR rats displayed disordered estrous cycles; decreased primordial follicles, primary follicles, and secondary follicles; increased atretic follicles; and differential expression of miRNAs when compared with normal rats. Given the contribution of acupuncture to ovarian reserve (Tang et al., 2015; Li et al., 2017; Yang and Yang, 2020), we evaluated differences in miRNA expression. We explored the effect of upstream miRNAs on diverse biological processes in DOR rats to elucidate the molecular mechanism underlying acupuncture treatment in TG-induced DOR rats. The results revealed that acupuncture might alter miRNA expression and further delay the decline in ovarian reserve.

As a vital component of traditional Chinese medicine, acupuncture has a curative effect on the body by stimulating the acupoints of meridians while inducing minimal side effects (Tang et al., 2015). Growing evidence has indicated that acupuncture participates in the therapeutic process of several diseases by attenuating oxidative stress (Peng et al., 2020; Yeung et al., 2021; Zhao et al., 2022). Oxidative stress is well known to play a critical role in the decline of ovarian function (Shi et al., 2016), inducing oocyte maturation deficiency (Shang et al., 2021), granulosa cell damage (Shen et al., 2017), decrease in mitochondrial DNA copy number, and telomere shortening (He et al., 2021), with oxidative lipid, protein, and DNA damage detected in aging ovaries, accompanied by a reduced antioxidant capacity (Lim and Luderer, 2011). Importantly, our previous studies reported that TGs could induce a high level of oxidative stress, and acupuncture could delay the progression of DOR in rats by suppressing oxidative stress and elevating anti-oxidation ability (Fang et al., 2019; Wang et al., 2021). Accordingly, we focused on the DE miRNAs associated with oxidative stress.

Over the last few decades, sequencing has been widely employed to investigate the underlying mechanisms and diagnosis of diseases (Baulina et al., 2022; Jiang et al., 2022; Quintanilla et al., 2022). Preliminary studies have revealed the role of miRNAs in the pathogenesis of DOR. For instance, miRNA sequencing of ovarian granulosa cells from women with DOR and those with normal ovarian reserves revealed 70 DE miRNAs; these differences were age-related (Liu et al., 2020a). A similar study was performed using mice (Kim et al., 2021a). In addition, 63 upregulated and 20 downregulated miRNAs were detected in 4-vinyl cyclohexene diepoxide-induced POF rats. Of these, miR-27b and miR-190 are associated with dysfunction of hormonal stimulus in the ovaries and may contribute to POF initiation, miR-151 and miR-672 regulate cell apoptosis, and miR-29a and miR-144 are involved in prostaglandin biosynthesis (Kuang et al., 2014). In the present study, 14 upregulated and 8 downregulated DE miRNAs were detected between DOR and CON rats, indicating that miRNAs were altered in DOR rats. Nevertheless, acupuncture modulated the expression of miRNAs, demonstrating 14 upregulated and 14 downregulated miRNAs. These results imply that acupuncture treatment could affect the expression of miRNAs in DOR rats.

To further explore the mechanism through which acupuncture regulates the ovarian reserve *via* miRNAs, GO and KEGG analyses were performed for functional enrichment analysis. Based on GO results, the target genes of DE miRNAs were mainly associated with biological processes, oxidation–reduction processes, multicellular organism development, and regulation of transcription by RNA polymerase. The KEGG pathway analysis suggested that the effect of acupuncture was primarily related to the mTOR signaling pathway, MAPK signaling pathway, and proteoglycans in cancer. miRNA sequencing of ovarian granulosa cells from women with DOR and those with normal ovarian reserve also revealed 70 DE miRNAs involved in the PI3K-Akt, MAPK, phospholipase D, and chemokine pathways. These differences are age-related (Liu et al., 2020a), consistent with our results.

It is well known that miRNA is a type of small non-coding RNA regulating more than 50% of the protein-coding genes (Tu et al., 2019). Mature miRNAs bind through imperfect complementary sequences in the 3-UTRs of target mRNAs as post-transcriptional regulators. Therefore, an individual miRNA can regulate hundreds of different mRNAs; conversely, multiple miRNAs can also target a single mRNA. A previous study confirmed that modulating the balance between oxidation and reduction mediates the ameliorative effects of acupuncture on DOR (Fang et al., 2019). Consequently, we constructed GO-mRNA and KEGG-mRNA networks for oxidative stress. In the GO-mRNA network, the top 10 target genes involved in the oxidation-reduction process network were oxidoreductases. Hsd17b11 and tecrl regulate oxidoreductase activity by acting on the CH-CH group of donors; hsd17b11 and srd5a3 act as NAD and NADP acceptors, respectively, while Rnls catalyzes NADP(+) formation. Notably, ptgs2 participates in oocyte maturation, ovulation, and the regulation of apoptotic processes (Park et al., 2021; Smith et al., 2022). Mdh2 regulates mitochondrial respiration and proliferation of ovarian cells (Pei et al., 2022). In the KEGG-mRNA network, the analysis was mostly focused on the mTOR pathway. mTOR signaling is a ROS-dependent pathway (Zhang et al., 2022) and may be involved in apoptosis and autophagy (Liu et al., 2020b). In addition, mTOR signaling plays a role in oocyte growth and granulosa cell regulation (Li and Liu, 2018). The mTOR pathway has been reported to protect granulosa cells from oxidative stress (Shen et al., 2017). mTOR has two forms of complexes: a highly conserved serine/threonine protein kinase, mTORC1, and mTORC2. mTORC1 is known to be involved in autophagy, oxidative stress, and protein synthesis. Among the 10 target genes of the mTOR pathway, Rnf152, Slc38a9, and Cab39 participate in mTOR regulation; of these, Slc38a9 acts as a lysosomal amino acid transporter in mTORC1 activation (Wyant et al., 2017), and Cab39 regulates mTOR via LKB-AMPK in an energy-dependent manner (Kuwabara et al., 2015). These results indicate that acupuncture might regulate some key oxidoreductase mRNAs via miRNAs in DOR rats.

We selected six DE miRNAs with | log2 (fold change) | ≥1 for validation. The six DE miRNAs overlapped between DOR versus CON and ACU versus DOR. These results differed slightly from those of miRNA sequencing. Among the six miRNAs, only rno-miR-92b-3p, mdo-miR-26b-5p_R+1_1ss10TC, and bta-miR-7857-3p_R-1 were significantly transcribed after acupuncture intervention. Compared with our results, data from other studies, including human and animal models, showed an overlap of DE miRNAs, in which mmu-miR-26a-5p expression was inversely proportional to the age of mice, and miR-92a-3p was increased in granulosa cells of women with DOR (Woo et al., 2018; Kim et al., 2021a).

## 5 Conclusion

In summary, the results of the present study suggest that acupuncture protected the ovarian reserve function of DOR rats by regulating the miRNA profile, which was probably related to the alleviation of oxidative stress. DE miRNAs, especially rno-miR-92b-3p, mdo-miR-26b-5p_R+1_1ss10TC, and bta-miR-7857-3p_R-1, might affect the oxidation–reduction process and mTOR pathway. This finding may provide crucial insights into the diagnosis and treatment of DOR. There are several limitations to the present study. First, we only verified miRNA transcription, and the overexpression strategy should be employed to investigate detailed mechanisms. Second, miRNA sequencing was performed on total ovarian tissue. Studies focusing on granulosa cells and oocytes are necessary to further explore the underlying mechanism of acupuncture in regulating ovarian reserves.

## Data Availability

The datasets presented in this study can be found in online repositories. The name of the repository and accession number are NCBI; PRJNA851266.
