# Supplementary material for: Effects of acupuncture treatment on microRNAs expression in ovarian tissues from Tripterygium glycoside-induced diminished ovarian reserve rats
Source: Front Genet. 2022 Sep 21;13:968711. doi: 10.3389/fgene.2022.968711 (PMC9532950; doi:10.3389/fgene.2022.968711)
Supplement: Supplementary file 2 [file Table1.docx]

**Supplementary Material**

**The method of the randomized digital table:** First, 18 rats were marked with the numbers 1-18 on the tails. Second, a randomized digital table was prepared (**Supplementary Table1**), and 18 randomized numbers were copied. Then divide these numbers by 3 and use the remainder 1, 2, and 3 to represent CON, DOR, and ACU groups respectively.

**Supplementary Table1** Randomized digital table

|  | **1** | **2** | **3** | **4** | **5** | **6** | **7** | **8** | **9** | **10** | **11** | **12** | **13** | **14** | **15** | **16** | **17** | **18** | **19** | **20** |
| --- | --- | --- | --- | --- | --- | --- | --- | --- | --- | --- | --- | --- | --- | --- | --- | --- | --- | --- | --- | --- |
| **1** | 16 | 55 | 72 | 86 | 44 | 67 | 60 | 93 | 34 | 28 | 94 | 55 | 13 | 74 | 71 | 22 | 43 | 31 | 97 | 7 |
| **2** | 40 | 99 | 93 | 87 | 41 | 92 | 25 | 92 | 27 | 2 | 76 | 18 | 88 | 34 | 55 | 17 | 59 | 79 | 13 | 9 |
| **3** | 23 | 70 | 59 | 17 | 79 | 90 | 42 | 55 | 72 | 36 | 69 | 54 | 42 | 22 | 40 | 55 | 43 | 54 | 15 | 83 |
| **4** | 25 | 88 | 61 | 2 | 33 | 42 | 47 | 56 | 33 | 75 | 8 | 91 | 42 | 99 | 45 | 43 | 72 | 28 | 59 | 12 |
| **5** | 24 | 13 | 69 | 51 | 56 | 31 | 43 | 83 | 46 | 3 | 26 | 1 | 37 | 40 | 70 | 69 | 35 | 54 | 32 | 5 |
| **6** | 45 | 6 | 68 | 98 | 56 | 67 | 58 | 15 | 20 | 36 | 58 | 96 | 57 | 61 | 98 | 12 | 15 | 98 | 63 | 42 |
| **7** | 20 | 67 | 29 | 66 | 68 | 27 | 25 | 15 | 74 | 25 | 21 | 93 | 53 | 10 | 66 | 35 | 73 | 54 | 11 | 86 |
| **8** | 58 | 81 | 26 | 59 | 67 | 9 | 16 | 9 | 87 | 36 | 58 | 93 | 1 | 84 | 82 | 43 | 40 | 8 | 69 | 46 |
| **9** | 25 | 13 | 99 | 62 | 99 | 56 | 14 | 61 | 28 | 87 | 1 | 16 | 46 | 68 | 92 | 16 | 61 | 22 | 73 | 61 |
| **10** | 65 | 10 | 37 | 81 | 7 | 72 | 60 | 88 | 85 | 60 | 3 | 73 | 58 | 79 | 78 | 56 | 54 | 92 | 43 | 42 |
| **11** | 88 | 87 | 12 | 84 | 77 | 91 | 44 | 23 | 63 | 8 | 68 | 16 | 32 | 74 | 17 | 95 | 71 | 89 | 78 | 62 |
| **12** | 59 | 34 | 59 | 91 | 21 | 82 | 9 | 72 | 63 | 43 | 67 | 8 | 69 | 50 | 73 | 25 | 2 | 81 | 74 | 86 |
| **13** | 95 | 18 | 88 | 2 | 76 | 71 | 7 | 6 | 32 | 72 | 77 | 74 | 24 | 78 | 62 | 40 | 69 | 96 | 62 | 68 |
| **14** | 40 | 11 | 40 | 70 | 39 | 12 | 36 | 32 | 55 | 92 | 66 | 29 | 30 | 30 | 71 | 15 | 52 | 39 | 5 | 18 |
| **15** | 45 | 9 | 87 | 95 | 88 | 20 | 11 | 4 | 2 | 78 | 48 | 46 | 92 | 61 | 51 | 19 | 97 | 18 | 55 | 10 |
| **16** | 22 | 64 | 12 | 11 | 73 | 32 | 82 | 14 | 43 | 90 | 68 | 82 | 69 | 87 | 72 | 37 | 23 | 91 | 45 | 69 |
| **17** | 55 | 54 | 12 | 24 | 91 | 70 | 60 | 83 | 91 | 8 | 13 | 98 | 33 | 13 | 62 | 24 | 70 | 54 | 99 | 78 |
| **18** | 45 | 28 | 62 | 15 | 95 | 5 | 58 | 78 | 50 | 68 | 38 | 51 | 62 | 35 | 63 | 37 | 32 | 80 | 30 | 21 |
| **19** | 74 | 35 | 24 | 89 | 22 | 91 | 37 | 7 | 64 | 67 | 12 | 50 | 54 | 11 | 28 | 95 | 13 | 52 | 40 | 15 |
| **20** | 25 | 56 | 96 | 22 | 86 | 49 | 56 | 48 | 5 | 24 | 66 | 86 | 56 | 39 | 86 | 57 | 95 | 57 | 96 | 46 |

**Supplementary Table2** Primers (miRNAs) used in qRT-PCR

| Gene (miRNAs) | Primer sequence (5’-3’) |
| --- | --- |
| U6 | Forward: CTCGCTTCGGCAGCACATATACT  Reverse: ACGCTTCACGAATTTGCGTGTC |
| U6 (stem-loop) | RT primer: GTCGTATCCAGTGCAGGGTCCGAGGTATTCGCACTGGATACGACAAAATA  Forward: AGAGAAGATTAGCATGGCCCCTG  Reverse: CAGTGCAGGGTCCGAGGT |
| rno-miR-92b-3p | TATTGCACTCGTCCCGGC |
| rno-miR-206-3p(stem-loop) | RT primer: GTCGTATCCAGTGCAGGGTCCGAGGTATTCGCACTGGATACGACCCACAC  Forward: GCGCGTGGAATGTAAGGAAGT  Reverse: AGTGCAGGGTCCGAGGTATT |
| mdo-miR-26-5p_R+1_1ss10TC | CCTTCAAGTAACCCAGGATAGGCT |
| bta-miR-7857-3p_R-1(stem-loop) | RT primer: GTCGTATCCAGTGCAGGGTCCGAGGTATTCGCACTGGATACGACAAGAGC  Forward: CGCGATTGTTCTCCAACCTG  Reverse: AGTGCAGGGTCCGAGGTATT |
| rno-miR-219a-2-3p_1ss10GC | CAGAATTGTGCCTGGACATCTGT |
| PC-3p-66859_94 | CCGCGTCGAACTTGACTATCTAGA |

**Supplementary Table 3** DE miRNAs in DOR rats

| **miRNA** | **Log2(fold-change)** | ***P*-value** | **Direction of regulation** | **Expression level** |
| --- | --- | --- | --- | --- |
| rno-miR-92b-3p | 1.51 | 0.001 | Up | Middle |
| mdo-miR-26-5p_R+1_1ss10TC | 0.98 | 0.004 | Up | Middle |
| rno-miR-206-3p | inf | 0.007 | Up | Middle |
| pal-miR-9993b-3p_1ss6GA | inf | 0.011 | Up | Middle |
| bta-miR-7857-3p_R-1 | inf | 0.015 | Up | Middle |
| eca-miR-8986b_L+1R-1_3ss2TC3GA21GA | 3.44 | 0.020 | Up | Middle |
| rno-miR-219a-2-3p_1ss10GC | inf | 0.022 | Up | Middle |
| pal-miR-9993a-3p_L+2_3 | 2.97 | 0.036 | Up | Middle |
| pal-miR-9993a-3p_L+2_4 | 2.97 | 0.036 | Up | Middle |
| pal-miR-9993a-3p_L+2_1 | 2.97 | 0.036 | Up | Middle |
| pal-miR-9993a-3p_L+2_2 | 2.97 | 0.036 | Up | Middle |
| mmu-mir-3968-p5_1ss10AT | inf | 0.040 | Up | Middle |
| PC-5p-6478_1795 | 0.61 | 0.046 | Up | Middle |
| PC-3p-66859_94 | 2.26 | 0.049 | Up | Middle |
| rno-miR-122-5p | -inf | 0.000 | Down | Middle |
| rno-miR-335_R-2 | -0.72 | 0.026 | Down | Middle |
| rno-miR-133a-3p_L-1R+1 | -0.27 | 0.028 | Down | Middle |
| rno-miR-24-1-5p_L+1R-1 | -0.46 | 0.029 | Down | Middle |
| rno-miR-129-2-3p | -1.07 | 0.034 | Down | Middle |
| rno-let-7d-3p | -0.28 | 0.038 | Down | High |
| mmu-miR-744-5p_R-1 | -0.43 | 0.040 | Down | High |
| rno-miR-664-2-5p_R+1 | -0.61 | 0.045 | Down | Middle |

**Supplementary Table 4** DE miRNAs in DOR rats after acupuncture treatment

| **miRNA** | **Log2(fold-change)** | ***P*-value** | **Direction of regulation** | **Expression level** | **Number of target genes** |
| --- | --- | --- | --- | --- | --- |
| rno-let-7e-3p | 0.25 | 0.008 | Up | Middle | 51 |
| pal-miR-9226-5p_L-4_1ss7CT | inf | 0.008 | Up | Middle | 1320 |
| rno-miR-451-5p_R-1 | 5.69 | 0.026 | Up | High | 129 |
| rno-miR-486 | 4.18 | 0.029 | Up | High | 541 |
| rno-miR-142-5p_L+2R-3 | 1.47 | 0.030 | Up | Middle | 542 |
| rno-miR-144-5p_R-1 | inf | 0.032 | Up | Middle | 295 |
| cgr-miR-486-5p_R+1 | 3.07 | 0.032 | Up | Middle | 544 |
| rno-miR-375-3p | inf | 0.034 | Up | Middle | 169 |
| rno-miR-3594-3p_L+2R-3 | 2.36 | 0.035 | Up | Middle | 803 |
| rno-miR-664-2-5p_R+1 | 0.83 | 0.038 | Up | Middle | 1366 |
| pal-miR-9993a-3p_L+1_1ss12GA | 1.19 | 0.040 | Up | Middle | 147 |
| rno-miR-486_L-1 | inf | 0.042 | Up | Middle | 663 |
| rno-miR-144-3p | inf | 0.043 | Up | Middle | 821 |
| bta-miR-2478_L-1_1ss11TC | 1.07 | 0.047 | Up | Middle | 675 |
| rno-miR-471-3p_R+2 | -1.16 | 0.002 | Down | Middle | 932 |
| rno-miR-92b-3p | -1.50 | 0.004 | Down | Middle | 522 |
| rno-miR-206-3p | -inf | 0.007 | Down | Middle | 914 |
| mdo-miR-26-5p_R+1_1ss10TC | -1.64 | 0.008 | Down | Middle | 804 |
| bta-miR-7857-3p_R-1 | -inf | 0.015 | Down | Middle | 706 |
| rno-miR-3580-3p | -0.54 | 0.019 | Down | Middle | 465 |
| rno-miR-219a-2-3p_1ss10GC | -inf | 0.022 | Down | Middle | 407 |
| rno-miR-450b-5p_R+1 | -0.86 | 0.031 | Down | High | 82 |
| PC-3p-66859_94 | -3.16 | 0.036 | Down | Middle | 121 |
| rno-miR-184 | -1.34 | 0.038 | Down | Middle | 285 |
| rno-miR-450a-5p | -0.82 | 0.039 | Down | High | 81 |
| rno-miR-742-5p_R+1 | -3.87 | 0.039 | Down | Middle | 886 |
| rno-miR-450a-3p_L-1R+2 | -1.08 | 0.041 | Down | Middle | 884 |
| hsa-mir-652-p5_1ss2CG | -2.37 | 0.044 | Down | Middle | 944 |
